# A rare case of endoscopically removing a fractured pancreatic stent related to surgery

**DOI:** 10.1055/a-2760-9250

**Published:** 2026-01-16

**Authors:** Jiangfeng Hu, Xingya Guo, Weiliang Jiang, Xiaobo Cai

**Affiliations:** 1 12482 Department of Gastroenterology, Shanghai General Hospital, Shanghai Jiaotong University, School of Medicine, Shanghai, China

**Fig. 1:**
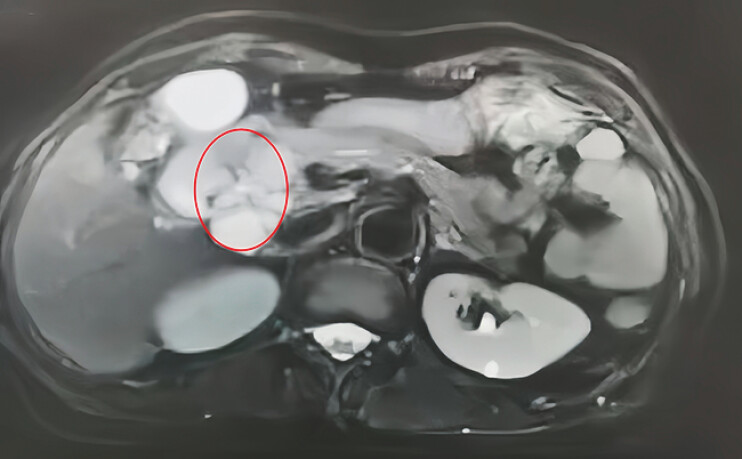
A MRI scan revealed a mass in the pancreatic head, suggesting a serous cystadenoma. MRI, magnetic resonance imaging.

**Fig. 2:**
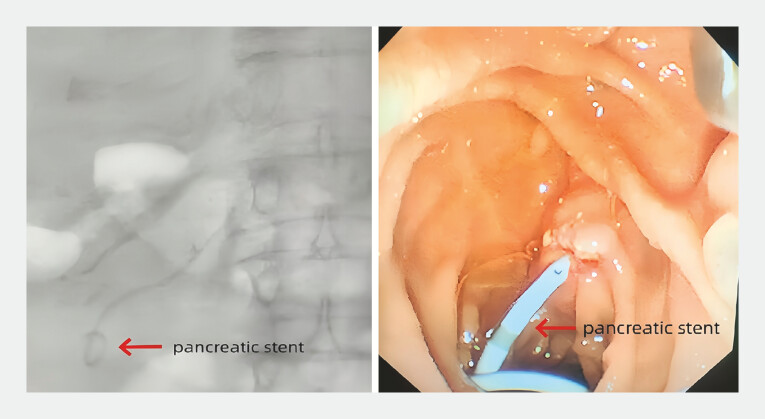
A single pigtail stent was placed within the pancreatic duct.

**Fig. 3:**
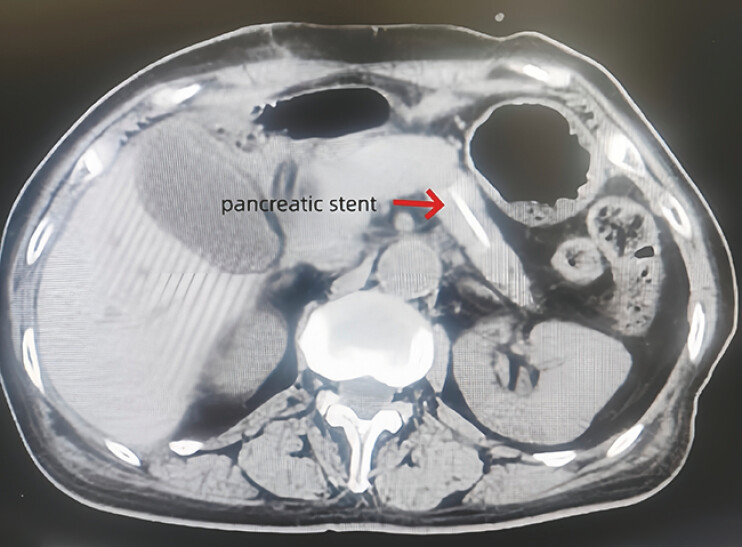
The pancreatic duct stent had shifted to the tail of the pancreas, and its length had significantly shortened.

**Fig. 4:**
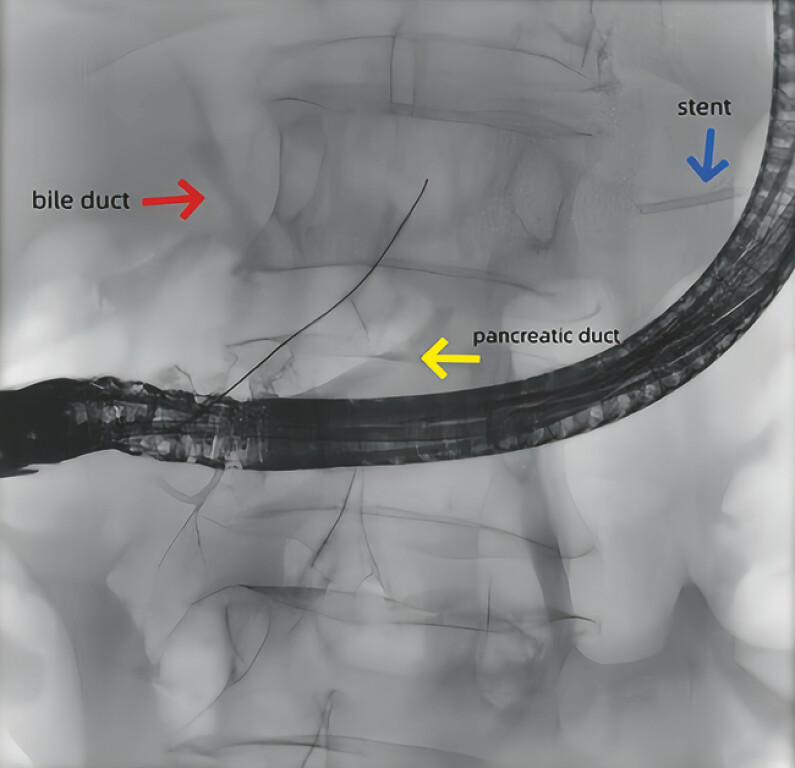
Pancreatography demonstrated stenosis and deformity of the pancreatic duct at the neck, along with contrast extravasation and opacification of the bile duct, increasing the possibility of a pancreaticobiliary fistula.

**Fig. 5:**
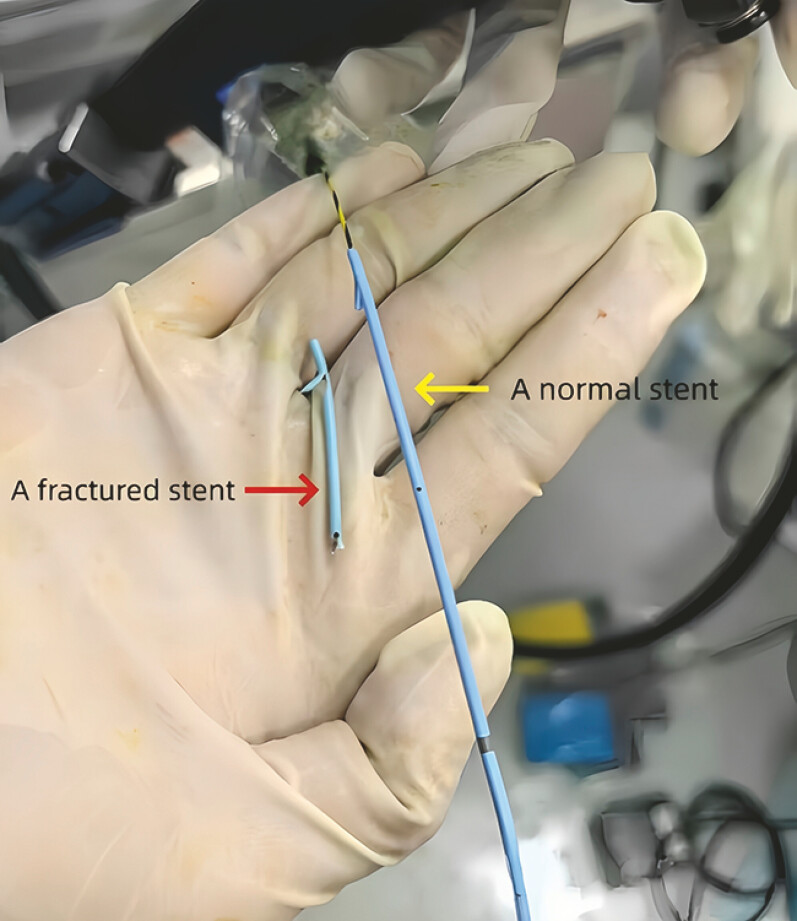
The fractured stent and the normal stent.

A 72-year-old woman underwent local resection for a pancreatic serous cystadenoma ( Fig. 1 ). A single-pigtail pancreatic duct stent (Micro-Tech, 5 Fr*7cm) was placed preoperatively via endoscopic retrograde cholangiopancreatography (ERCP; Fig. 2 ). Subsequently, she underwent local pancreatic resection and T-tube placement in the bile duct. Three months postoperatively, a computed tomographic (CT) scan revealed that the stent had migrated to the tail of the pancreas and appeared significantly shortened, suggesting stent fracture ( Fig. 3 ). A subsequent ERCP was performed for stent retrieval. Pancreatography demonstrated stenosis and deformity of the pancreatic duct at the neck, along with contrast extravasation and opacification of the bile duct, increasing the possibility of a pancreaticobiliary fistula. After difficult cannulation with a standard guidewire, a super-fine curved guidewire (Olympus VisiGlide 2) was successfully advanced into the pancreatic duct ( Fig. 4 ). The stenotic segment was dilated with a 4 mm × 6 cm balloon (Hurricane, Boston Scientific). A cholangioscope (EyeMAX, 9 Fr) was then introduced, traversing the narrowing and identifying the fractured stent fragment within the pancreatic duct. The retained stent was successfully grasped under direct vision using SpyBite MAX biopsy forceps (Boston Scientific) and retrieved along with the cholangioscope ( Video 1 , Fig. 5 ). A new stent was placed in the pancreatic duct. The patient had an uneventful recovery and was discharged 3 days postoperatively.

A rare case of endoscopically removing a fractured pancreatic stent related to surgery.Video 1

Stent migration within the pancreatic duct is rare, with a reported incidence of approximately 0.2%. Stent fracture is also uncommon; one study reported an incidence of 1.2%, although all cases involved straight stents that fractured during removal due to pancreatic duct stenosis 1 2 . This is the first reported case of a surgical-related pancreatic duct stent fracture with proximal migration. The presence of pancreatic duct distortion and stenosis rendered the retrieval procedure particularly challenging. The use of cholangioscopy-guided intervention proved effective for the successful and safe removal of the migrated stent fragment.

Endoscopy_UCTN_Code_CPL_1AK_2AD
